# Neurocircuitry Underlying Stress and Emotional Regulation in Animals Prenatally Exposed to Alcohol and Subjected to Chronic Mild Stress in Adulthood

**DOI:** 10.3389/fendo.2014.00005

**Published:** 2014-02-13

**Authors:** Charlis Raineki, Kim G. C. Hellemans, Tamara Bodnar, Katie M. Lavigne, Linda Ellis, Todd S. Woodward, Joanne Weinberg

**Affiliations:** ^1^Department of Cellular and Physiological Sciences, The University of British Columbia, Vancouver, BC, Canada; ^2^Department of Neuroscience, Carleton University, Ottawa, ON, Canada; ^3^Department of Psychiatry, The University of British Columbia, Vancouver, BC, Canada; ^4^BC Mental Health and Addictions Research Institute (BCMHARI), Vancouver, BC, Canada

**Keywords:** prenatal alcohol exposure, chronic mild stress, amygdala, hippocampus, prefrontal cortex, paraventricular nucleus of hypothalamus, *c-fos*, constrained principal component analysis

## Abstract

Individuals exposed to alcohol during gestation show higher rates of psychopathologies. The hyperresponsivity to stress induced by prenatal alcohol exposure (PAE) may be related to this increased rate of psychopathologies, especially because this population is more likely to be exposed to stressful environments throughout life. However, alcohol-induced changes in the overlapping neurocircuitries that underlie stress and the expression of psychopathologies are not fully understood. Here, we performed a comprehensive analysis of the neural activity within central areas known to play key roles in both emotional and stress regulation. Adult male and female offspring from PAE, pair-fed, and *ad libitum*-fed control conditions were exposed to chronic mild stress (CMS). Following CMS, the neural activity (*c-fos* mRNA) of the amygdala, ventral hippocampal formation, medial prefrontal cortex (mPFC), and paraventricular nucleus of hypothalamus (PVN) was assessed in response to an acute stress (elevated plus maze). Our results demonstrate that, overall, PAE decreased neural activity within the amygdala and hippocampal formation in males and increased neural activity within the amygdala and mPFC in females. CMS reduced neural activity within the mPFC and PVN in PAE males, but reduced activity in all areas analyzed in control males. By contrast, CMS reduced neural activity in the mPFC in PAE females and had no effects in control females. Furthermore, the constrained principal component analysis revealed that these patterns of neural activity resulted in differential activation of the functional neural networks in males compared to females, indicating sexually dimorphic effects of PAE and CMS. Importantly, the altered networks of brain activation in PAE animals may underlie the hyperresponsivity to stress and increased psychopathologies observed among individuals prenatally exposed to alcohol.

## Introduction

Fetal development is a dynamic process strongly influenced by the quality of the environment in which it occurs (1, 2). Adverse intrauterine environments can negatively alter the developmental trajectory, resulting in physical and mental health problems later in life (1, 3). In mammals, the mother plays a critical role in fetal development, not only by supplying nutrients and oxygen via the placenta, but also by modulating the environmental stimuli to which the fetus is exposed (4). Clinical and experimental studies have clearly demonstrated that alcohol consumed during pregnancy has pervasive and long-lasting negative effects on fetal development (5–9). Indeed, prenatal alcohol exposure (PAE) is strongly associated with a wide range of neural, behavioral, hormonal, and cognitive deficits in humans, non-human primates, and rodents (5–9). In addition, the rates of psychopathologies (e.g., anxiety, depression, other mood disorders, and substance use disorders) among individuals prenatally exposed to alcohol are disproportionately higher when compared to unexposed individuals (10–13). Unfortunately, little is known about the underlying neural processes that support this high incidence of psychopathologies following PAE.

Brain areas implicated in psychopathologies overlap to a large extent with areas that mediate responses to stress. The amygdala, hippocampus, and medial prefrontal cortex (mPFC) are intrinsically involved in the modulation of hypothalamic–pituitary–adrenal (HPA) axis activity (14, 15), and are also associated with several mental health disorders (16, 17). Abnormal function of any one of these highly interconnected areas may result in abnormal responses to stress and/or the emergence of psychopathologies. This is especially relevant for individuals exposed to alcohol during gestation, as neuroimaging studies have demonstrated structural and functional alterations in brain regions involved in stress regulation, such as the hippocampus and PFC (18–21).

Not surprisingly, a striking deficit induced by PAE involves changes in how affected individuals process and/or respond to a stressful stimulus or situation. The human and animal literature clearly demonstrates that alcohol consumption during pregnancy results in offspring that are hyperresponsive to a variety of stressors (5, 9, 22–24). For example, children exposed to alcohol *in utero* show higher salivary cortisol levels following exposure to stressors such as blood draw and the still-face procedure when compared to unexposed counterparts (22, 23). Additionally, higher basal cortisol levels were also observed in children exposed to alcohol prenatally (23, 24). In parallel with these studies, rats prenatally exposed to alcohol show increased HPA activation and/or a delay in return to basal levels as compared to controls in response to a wide range of stressful stimuli including footshock, restraint, immune challenge, and exposure to novel environments (25–30). In addition, HPA dysregulation is observed not only following stress, but also under basal conditions, even in the face of similar basal hormone levels. Dysregulation is evident at multiple levels of the axis, and appears to reflect changes in both HPA drive and feedback regulation and/or in the balance between drive and feedback. Taken together, these findings suggest that for individuals prenatally exposed to alcohol, stressors occurring over the life span may be acting on an already dysregulated or sensitized stress neurocircuitry. Thus, stress hyperresponsivity and HPA dysregulation may be a crucial factor mediating the increased vulnerability of these individuals to develop later psychopathologies (5, 31, 32). An aggravating factor to this already unfavorable situation is that individuals prenatally exposed to alcohol are, in general, at a higher risk of encountering a more stressful environment throughout the lifespan (12, 13, 33).

Using a rodent model of PAE, our laboratory has been successful in demonstrating that the combination of PAE and chronic unpredictable stress in adulthood increases anxiety- and depressive-like behaviors in a sexually dimorphic manner (5, 31, 32). However, the neurocircuitry underlying both stress dysregulation and anxiety- and depressive-like behaviors of individuals prenatally exposed to alcohol is still not fully understood. In the present study, adult male and female rats that were prenatally exposed to alcohol underwent a 10-day chronic mild stress (CMS) regimen in adulthood, which was followed by a comprehensive analysis of the neural activity within brain areas known to play key roles in stress and emotional regulation. Specifically, we assessed *c-fos* mRNA expression as a measurement of neural activity in the medial parvocellular dorsal division of the paraventricular nucleus of hypothalamus (mpdPVN), ventral hippocampal formation (CA1, CA3, DG, and ventral subiculum), the amygdala (central, cortical, lateral, basal, and medial nuclei), and the mPFC [anterior cigulate cortex (ACC), prelimbic (PrL), and infralimbic (IL)] in response to an acute stressor – exposure to the elevated plus maze (34, 35). In order to identify networks of coordinated activity in these brain regions rather than simply to identify whether activity differed between experimental groups for each individual brain area, we employed constrained principal component analysis (CPCA) in addition to a traditional univariate analysis (ANOVAs followed by *post hoc* group comparisons). CPCA is a multivariate technique that combines multivariate multiple regression and principal component analysis (PCA) into a unified framework (36–41). In the current study, CPCA allowed for the identification of brain networks that were specifically related to our experimental conditions (i.e., PAE and CMS). Thus, we were able to identify the networks of brain regions associated with differences in neural activity between animals that were prenatally exposed to alcohol, with or without CMS exposure in adulthood, and their respective controls. Below, we present results from a traditional univariate technique (ANOVA) as well as CPCA to demonstrate how this multivariate technique can facilitate interpretation when group differences are observed in a variety of distinct, but interconnected, brain regions.

## Materials and Methods

### Animals and breeding

Female Sprague-Dawley rats were obtained from Charles River Laboratories (St. Constant, QC, Canada) and male Sprague-Dawley rats were obtained from the UBC Animal Care Centre. Rats were pair-housed by sex and maintained at a constant temperature (21 ± 1°C) and on a 12-h light–dark cycle (lights on at 6 a.m.) with *ad libitum* access to water and standard laboratory chow (Jamieson’s Pet Food Distributors Ltd., Canada). After a 10-day acclimation period, male and female pairs were placed together suspended in stainless steel cages with mesh front and floor (25 cm × 18 cm × 18 cm). Day 1 of gestation (G1) was indicated by the presence of a vaginal plug on the wax paper beneath the breeding cages, which were checked daily. All experiments were performed in accordance with National Institutes of Health (NIH) guidelines for the care and use of laboratory animals and the Canadian Council on Animal Care guidelines and were approved by The University of British Columbia Animal Care Committee.

### Prenatal alcohol exposure

On G1, females were single-housed and randomly assigned to one of the three prenatal treatment groups: PAE, Pair-Fed (PF), or *ad libitum*-fed Control (C). Dams in the PAE group were offered *ad libitum* liquid ethanol diet with 36% ethanol-derived calories (Dyets, Inc., Bethlehem, PA, USA). This diet is formulated to provide adequate nutrition to pregnant rats regardless of ethanol intake (42, 43). PF dams were offered a liquid control diet with maltose–dextrin isocalorically substituted for ethanol, in an amount matched to the consumption of a PAE partner according to gestation day (gram/kilogram body weight/day of gestation). The control dams were offered *ad libitum* access to standard laboratory chow (Jamieson’s Pet Food Distributors Ltd.). All animals were provided with fresh diet daily within 1 h of lights off to prevent a shift in corticosterone circadian rhythms, which occurs in animals that are on a restricted feeding schedule, such as the PF dams (44, 45). Experimental diets were continued through G21. Beginning on G22, all animals were offered *ad libitum* access to standard laboratory chow and water, which they received throughout lactation. Pregnant dams were left undisturbed except for cage changing and weighing, which occurred on G1, G7, G14, and G21. On the day of birth [postnatal day 1 (PN1)], litters were weighed and culled to 10 pups with an attempt to preserve an equal number of males and females per litter. Dams and pups were weighed on PN1, PN8, PN15, and PN22 [dam and pup body weight data were published in Ref. (31)]. On PN22, pups were weaned and group-housed by litter and sex.

### Chronic mild stress

On PN40, one male and one female rat from each litter were randomly assigned to either the CMS or non-CMS condition and were pair-housed with another animal of the same sex, prenatal treatment, and stress condition (CMS or non-CMS). In adulthood (PN60–90), CMS animals were subjected to 10 consecutive days of randomized stressors. Animals were exposed to two different stressors each day: the first at a randomized time in the morning (between 8:00 and 12:00 h) and the second at a randomized time in the afternoon (between 13:00 and 16:00 h), with a minimum of 2 h between stressors. On day 1 of CMS, all animals were weighed, handled, and moved to new colony rooms in a neighboring building. CMS and non-CMS animals were housed in separate colony rooms so that non-CMS animals were not exposed to the disturbance of either moving CMS animals between stressors, or stress odors and vocalizations produced by CMS animals. CMS exposure occurred in a room separate from the colony room. The order and type of stressor were randomized, but all animals received the same number of exposures to each stressor over the 10-day period. Stressors included: (1) platform: 5 min exposure to an elevated Plexiglass platform (20 cm × 20 cm) mounted on a 90-cm high post; (2) cage tilt: the home cage was tilted at a 30° angle for 2 h; (3) novel cage: exposure to a novel, small (18 cm × 25 cm × 15 cm), and opaque cage without food and water for 1 h; (4) soiled cage: exposure to the soiled cage from another sex-matched pair of animals for 1 h; (5) restraint: restraint in PVC tubes (15 cm × 6 cm for females and 19 cm × 7 cm for males) for 30 min; (6) social isolation: overnight isolation in hanging wire mesh cage (20 cm × 23 cm × 18 cm) without food and water for 12 h; (7) white noise: exposure to white noise (40 dB; Lafayette Instruments model no. 15800) for 2 h; and (8) tail nick: a cut was made 1 mm from the tip of the tail for blood collection from the tail vein [blood sample data was published in Ref. (31)]. Non-CMS animals remained undisturbed other than routine husbandry during this same period.

### Acute stress (elevated plus maze exposure) and brain collection

The day after the end of CMS (day 11), all animals from both CMS and non-CMS conditions were habituated to the behavioral testing room for 10 min. The following day (day 12), animals were exposed to an elevated plus maze for 5 min in a dimly lit room during the light phase (09:00 and 12:00 h) of the light–dark cycle [elevated plus maze data were published in Ref. (31)]. Exposure to the elevated plus maze is a stressor known to produce an increase in corticosterone levels (35). The elevated plus maze consisted of two closed arms (69 cm × 10.5 cm) and two open arms (69 cm × 10.5 cm) with a central platform (diameter 35 cm). Closed arms had walls of darkened Plexiglass 20 cm high along their length. Open arms had a 2-cm-high lip along the edges of the arms. Immediately following the elevated plus maze exposure, animals were individually housed and left undisturbed in a quiet holding room for 30 min. Animals were then decapitated, and brains were removed, frozen on dry ice, and stored at −80°C.

### Neural assessment of *c-fos* mRNA by *In situ* hybridization

#### Probes and labeling

The ribonucleotide probe was prepared using a rat *c-fos* 2116 bp template provided by Dr. Victor Viau (Department Cellular and Physiological Sciences, The University of British Columbia, Canada). Probes were labeled with ^35^S-UTP (Amersham Biosciences, NJ, USA) using Polymerase T^7^ and Promega Riboprobe System (Promega Corporation, Madison, WI, USA). All probes were purified using Micro Bio-Spin 30 Columns (Bio-Rad, CA, USA). One molar of DTT was added to prevent oxidation.

#### *In situ* hybridization

Brains were sectioned coronally (20 μm) using a cryostat (−16°C) and stored at −80°C. Thawed sections were fixed in formalin for 30 min and then pre-hybridized as follows: 1× PBS twice for 10 min each, proteinase K (100 μg/L; at 37°C) for 9 min, 0.1 M triethanolamine-hydrochloride (TEA) for 10 min, 0.1 M TEA with 0.25% acetic anhydride for 10 min, 2× SSC twice for 10 min each, dehydration by a graded series of ethanol, chloroform for 5 min, and finally 100% ethanol before being air dried. Hybridization buffer (75% formamide, 3× SSC, 1× Denhardt’s solution, 200 μg/mL yeast tRNA, 50 mM sodium phosphate buffer (pH 7.4), 10% dextran sulfate, and 10 mM DTT) was applied (1 × 10^6^ cpm/slide) and covered with HybriSlips (Sigma-Aldrich, ON, Canada). Sections were incubated overnight at 55°C in chambers humidified with 75% formamide. HybriSlips were removed and the slides were rinsed as follows: 2× SSC twice for 20 min, 2× SSC for 30 min, 50 μg/L RNAse A solution (at 37°C) for 60 min, 2× SSC with 0.01 M DTT for 10 min, 1× SSC for 10 min, 0.5× SSC with 0.01 M DTT for 10 min, 0.1× SSC with 0.01 M DTT (at 60°C) for 60 min, and 0.1× SSC for 5 min. Sections were dehydrated by a graded series of ethanol and air dried overnight.

Kodak BioMax autoradiography film was exposed to hybridized slides of the ventral hippocampus for 28 days, and then developed using Kodak GBX developer and fixer. For all other areas, the hybridized slides were dipped in Kodak NTB2 autoradiography emulsion (diluted 50:50 with distilled water) and exposed in desiccated sealed, light tight boxes (4°C) for 49 days for mPFC, 70 days for mpdPVN, and 92 days for the amygdala. Slides were developed using Kodak D19 developer and standard Kodak fixer, counterstained with Toluidine Blue, and coverslipped with Permount (Fisher Scientific Ltd.). For all brain areas analyzed, *n* = 4–5 with the exception of the ventral hippocampal formation (CA1, CA3, DG, and ventral subiculum) where *n* = 3 for PF non-CMS males.

#### Densitometric analysis

The autoradiographic films for the ventral hippocampal formation were scanned and analyzed with Scion Image 4.0.3.2 (National Institutes of Health, USA). The left and right DG, CA1, and CA3, and ventral subiculum were traced freehand according to a stereotaxic rat brain atlas (46) in two sections per animal to determine mean gray density levels. Background was measured from the corpus callosum, and corrected gray levels were obtained by subtracting the background level from each of the four measurements. Left and right levels in each measured area were averaged together for analysis. For the emulsion dipped slides (amygdala, mPFC, and mpdPVN), *in situ* signals were visualized with a Q-imaging monochrome 12-bit camera attached to a Zeiss Axioskop 2 motorized plus microscope. Images were captured under dark field illumination using Northern Elite 6.0v (Empix Imaging, Inc., Mississauga, ON, Canada) and analyzed with ImageJ 10.3 software (National Institutes of Health, USA).

### Statistical analysis

**Figure 1:**
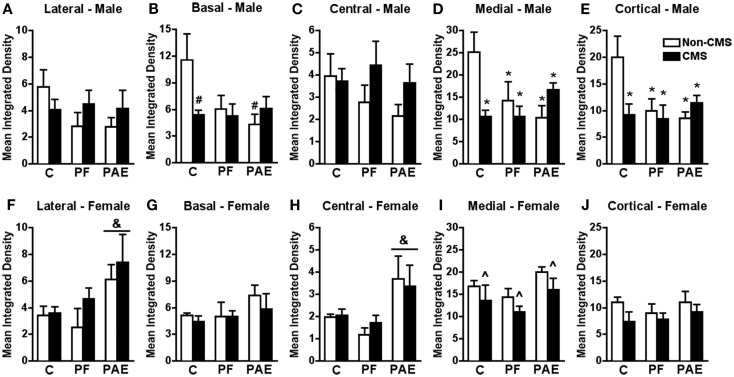
**Amygdala *c-fos* mRNA expression in response to the elevated plus maze test in male and female control (C), pair-fed (PF), and prenatal alcohol exposed (PAE) rats, with or without exposure to chronic mild stress (CMS) in adulthood**. Bars represent the integrated density (mean ± SEM) of *c-fos* mRNA expression in the lateral **(A,F)**, basal **(B,G)**, central **(C,H)**, medial **(D,I)**, and cortical **(E,J)** amygdala nuclei. *Indicates a significant interaction between prenatal treatment and CMS exposure where all groups are different from C non-CMS; ^&^indicates a significant main effect of prenatal treatment where the *post hoc* test shows that PAE is different from C and PF, independent of CMS; ^∧^indicates a significant main effect of CMS exposure where all animals exposed to CMS are different from animals not exposed to CMS; ^#^indicates that control CMS and PAE non-CMS are different from control non-CMS based on *a priori* comparisons (*n* = 4–5 for all groups).

**Figure 2:**
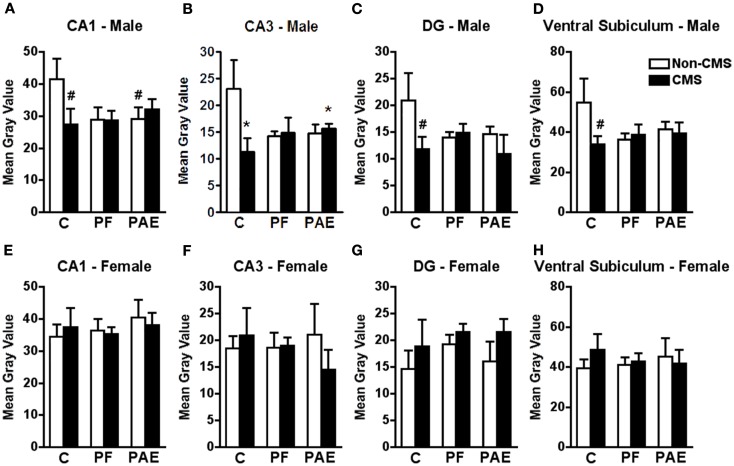
**Hippocampal formation *c-fos* mRNA expression in response to the elevated plus maze test in male and female control (C), pair-fed (PF), and prenatal alcohol exposed (PAE) rats, with or without exposure to chronic mild stress (CMS) in adulthood**. Bars represent the gray value (mean ± SEM) of *c-fos* mRNA expression in the CA1 **(A,E)**, CA3 **(B,F)**, DG **(C,G)**, and ventral subiculum **(D,H)**. *Indicates a significant interaction between prenatal treatment and CMS exposure, where the control non-CMS group is different from control CMS and PAE CMS groups; ^#^indicates that control CMS and PAE non-CMS are different from control non-CMS based on *a priori* comparisons (*n* = 3–5 for all groups).

**Figure 3:**
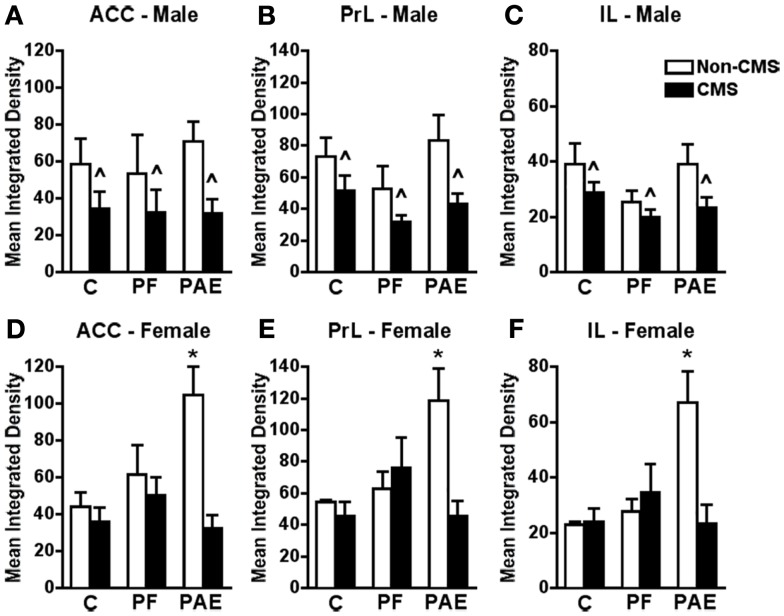
**Medial prefrontal cortex *c-fos* mRNA expression in response to the elevated plus maze test in male and female control (C), pair-fed (PF), and prenatal alcohol exposed (PAE) rats, with or without exposure to chronic mild stress (CMS) in adulthood**. Bars represent the integrated density (mean ± SEM) of *c-fos* mRNA expression in the ACC **(A,D)**, PrL **(B,E)**, and IL **(C,F)**. *Indicates a significant interaction between prenatal treatment and CMS exposure where the PAE non-CMS group is different from all other groups; ^∧^indicates a significant main effect of CMS exposure (*n* = 4–5 for all groups).

**Figure 4:**
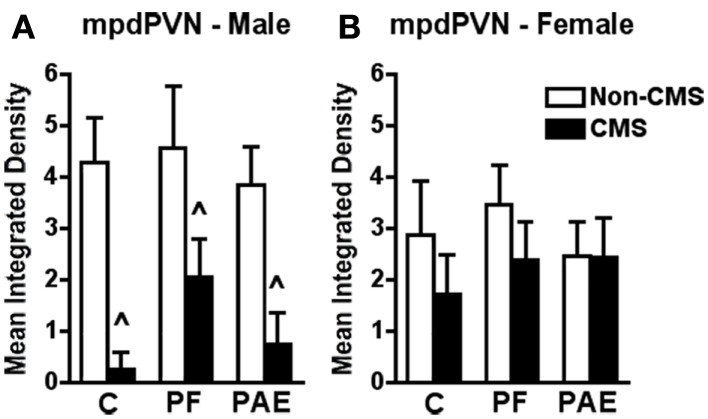
**Medial parvocellular dorsal division of the paraventricular nucleus of hypothalamus *c-fos* mRNA expression in response to the elevated plus maze test in male (A) and female (B) control (C), pair-fed (PF), or prenatal alcohol exposed (PAE) rats, with or without exposure to chronic mild stress (CMS) in adulthood**. Bars represent the integrated density (mean ± SEM) of *c-fos* mRNA expression in the mpdPVN. ^∧^Indicates a significant main effect of CMS exposure (*n* = 4–5 for all groups).

Data were first analyzed using two-way analysis of variance (ANOVA for the factors of prenatal treatment and CMS exposure) followed by Newman–Keuls *post hoc* tests for each brain region (presented in Figures 1–4). Differences were considered significant at *p* ≤ 0.05. Further analyses utilized planned comparisons to test the *a priori* hypotheses that: (1) PAE will alter the response to acute stress, i.e., non-CMS PAE animals will show differential neural activity in response to acute stress (exposure to the elevated plus maze) compared to non-CMS control animals; and (2) CMS will differentially alter neural activity in response to acute stress in PAE compared to control animals.

In order to identify networks of brain regions altered by our experimental conditions, we employed CPCA, which is performed in two steps, referred to as the external analysis and the internal analysis. The external analysis consists of multivariate least-squares multiple regression, which serves to separate the overall variance (i.e., *c-fos* mRNA activity in the 13 brain regions of interest) into the variance that can (and cannot) be predicted by the experimental conditions (i.e., prenatal treatment and CMS exposure). This first step of CPCA simply separates the predicted and residual scores through multivariate multiple regression, using dummy-coded groupings as the independent variables [1 for group membership, 0 for not; (36)]. This first step produces a matrix of predicted scores that reflects the portion of variance in brain neural activity that is attributable to the experimental conditions (i.e., the predictable variance). A matrix of residual scores is also produced, which reflects the portion of variance in brain neural activity that is not attributable to experimental conditions (i.e., the residual variance). This residual variance was not further analyzed for the present study, as we were interested in brain networks associated with our experimental conditions. The second step in CPCA, the internal analysis, simply consists of a PCA on the predictable variance (i.e., the predicted scores from the multivariate regression in the first step). PCA is a data reduction technique that serves to combine large sets of related variables by reducing them to a smaller number of components (referred to as networks when the dependent variables consist of brain regions) that best explain the variance, while losing as little information as possible. The components that emerge from the PCA on the predicted scores reflect the brain networks that characterize the differences in brain activity between the experimental conditions (i.e., the brain networks that show differing degrees of activity across the different prenatal treatment and CMS groups). All PCA solutions were separately rotated using Varimax with Kaiser normalization, and the number of the components extracted was determined by inspection of scree plots (47, 48). In order to examine specifically how the groups differ in terms of brain activity in each of the resulting networks, correlations were computed between the experimental groups and the component scores from each of the extracted components. A more detailed description of the theory and methodology of CPCA can be found in previously published manuscripts (38–41, 49, 50). CPCA was carried out using SPSS.

## Results

### Amygdala

#### Males

*c-fos* mRNA expression within the medial and cortical amygdala nuclei in response to acute stress was significantly reduced in non-CMS PAE and PF compared to non-CMS control males (Figures 1D,E). Moreover, while CMS reduced *c-fos* mRNA expression within the medial and cortical amygdala nuclei in control males compared to that in their non-CMS counterparts, *c-fos* mRNA expression of PAE and PF rats was similar under CMS and non-CMS conditions following acute stress {significant interaction between prenatal treatment and CMS exposure for medial [*F*_(1,23)_ = 6.578; *p* < 0.007] and cortical [*F*_(1,23)_ = 4.196; *p* < 0.03] amygdala nuclei}.

Similarly, *c-fos* mRNA expression within the basal amygdala in response to acute stress was significantly reduced in non-CMS PAE compared to non-CMS control males (Figure 1B). Moreover, CMS control males showed reduced *c-fos* mRNA expression within the basal nucleus compared to that in their non-CMS counterparts, whereas PAE rats were similar in *c-fos* mRNA expression following acute stress under both CMS and non-CMS conditions {no significant interaction between prenatal treatment and CMS interaction [*F*_(1,23)_ = 3.129; *p* = 0.06]; *a priori* analysis indicated that PAE non-CMS (*p* < 0.005) and control CMS (*p* < 0.02) showed lower *c-fos* mRNA expression than the control non-CMS}.

Neither prenatal treatment nor CMS significantly altered *c-fos* mRNA expression within the central or lateral amygdala nuclei following acute stress {Figures 1A,C; no significant interaction between prenatal treatment and CMS exposure for central [*F*_(1,22)_ = 0.803] and lateral [*F*_(1,23)_ = 1.499] amygdala nuclei}.

#### Females

Among females, a different pattern of neural activity within the amygdala was observed. PAE, independent of CMS exposure, increased *c-fos* mRNA expression within the central and lateral amygdala nuclei in response to acute stress when compared to that in control and PF animals {Figures 1F,H; no significant interaction between prenatal treatment and CMS exposure for central [*F*_(1,23)_ = 0.448] or lateral [*F*_(1,23)_ = 0.387] amygdala nuclei, but significant main effect of prenatal treatment for central [*F*_(2,23)_ = 7.198; *p* < 0.005] and lateral [*F*_(2,23)_ = 4.910; *p* < 0.02] nuclei in response to acute stress}.

In the medial amygdala, CMS reduced *c-fos* mRNA expression in response to acute stress independent of prenatal treatment group {Figure 1I; no significant interaction between prenatal treatment and CMS exposure [*F*_(1,23)_ = 0.015], but significant main effect for CMS [*F*_(1,23)_ = 4.176; *p* = 0.05]}.

Neither prenatal treatment nor CMS significantly altered *c-fos* mRNA expression in response to acute stress within the cortical and basal amygdala nuclei {Figures 1G,J; no significant interaction between prenatal treatment and CMS exposure for cortical [*F*_(1,23)_ = 0.312] or basal [*F*_(1,23)_ = 0.257] amygdala nuclei}.

### Hippocampal formation

#### Males

*c-fos* mRNA expression within the CA3 subregion of the ventral hippocampal formation in response to acute stress was significantly reduced in CMS PAE compared to non-CMS control males (Figure 2B). Moreover, similar to the medial and cortical amygdala, while CMS reduced *c-fos* mRNA expression within the CA3 region in control males compared to their non-CMS counterparts, *c-fos* mRNA expression of PAE and PF rats was similar under CMS and non-CMS conditions in response to acute stress {significant interaction between prenatal treatment and CMS exposure [*F*_(1,19)_ = 3.714; *p* < 0.05]}.

Consistent with these findings, *c-fos* mRNA expression in response to acute stress within the CA1 subregion of the ventral hippocampal formation was significantly reduced in non-CMS PAE compared to non-CMS control males (Figure 2A). Moreover, while *c-fos* mRNA expression was reduced within the CA1, DG, and ventral subiculum subregions in CMS control compared to non-CMS control males, *c-fos* mRNA expression of PAE rats was similar under CMS and non-CMS conditions in response to acute stress {Figures 2A,C,D; no significant interaction between prenatal treatment and CMS interaction for CA1 [*F*_(1,20)_ = 2.257], DG [*F*_(1,20)_ = 1.472], or ventral subiculum [*F*_(1,19)_ = 1.839]; *a priori* analysis indicates that non-CMS PAE showed a significantly lower *c-fos* mRNA expression within the CA1 (*p* = 0.05) than non-CMS controls, and that CMS controls (*p* < 0.03) showed lower *c-fos* mRNA expression than non-CMS control within CA1, DG, and ventral subiculum}.

#### Females

Among females, neither prenatal treatment nor CMS significantly altered *c-fos* mRNA expression in response to acute stress within any subregion of the hippocampal formation {Figures 2E–H; no significant interaction between prenatal treatment and CMS exposure for CA1 [*F*_(1,21)_ = 0.193], CA3 [*F*_(1,21)_ = 0.399], DG [*F*_(1,21)_ = 0.130], or ventral subiculum [*F*_(1,21)_ = 0.490]}.

### Medial prefrontal cortex

#### Males

In all mPFC subregions (ACC, PrL, and IL), CMS reduced *c-fos* mRNA expression in response to acute stress independent of prenatal treatment {Figures 3A–C; no significant interaction between prenatal treatment and CMS exposure for ACC [*F*_(1,22)_ = 0.299], PrL [*F*_(1,22)_ = 0.474], or IL [*F*_(1,22)_ = 0.421], but significant main effect for CMS for ACC [*F*_(1,22)_ = 7.426; *p* < 0.02], PrL [*F*_(1,22)_ = 8.936; *p* < 0.008], and IL [*F*_(1,22)_ = 5.365; *p* < 0.03]}.

#### Females

Similar to findings for the amygdala, *c-fos* mRNA expression in response to acute stress within all areas of the mPFC was significantly increased in non-CMS PAE females compared to non-CMS control and PF females (Figures 3D–F). Moreover, while CMS PAE females showed reduced *c-fos* mRNA expression within the ACC, PrL, and IL compared to non-CMS PAE females, *c-fos* mRNA expression of control and PF rats was similar under CMS and non-CMS conditions in response to acute stress {significant interaction between prenatal treatment and CMS exposure for ACC [*F*_(1,23)_ = 4.614; *p* < 0.03], PrL [*F*_(1,23)_ = 5.139; *p* < 0.02], and IL [*F*_(1,23)_ = 6.525; *p* < 0.007]}.

### Medial parvocellular dorsal division of the paraventricular nucleus of hypothalamus

#### Males

In the mpdPVN, CMS reduced *c-fos* mRNA expression in response to acute stress independent of prenatal treatment {Figure 4A; no significant interaction between prenatal treatment and CMS exposure [*F*_(1,23)_ = 0.257], but significant main effect for CMS [*F*_(1,23)_ = 15.541; *p* < 0.001]}.

#### Females

By contrast to males, neither prenatal treatment nor CMS significantly altered *c-fos* mRNA expression among females within mpdPVN in response to acute stress {Figure 4B; no significant interaction between prenatal treatment and CMS exposure [*F*_(1,23)_ = 0.783]}.

### Constrained principal component analysis

#### Males

**Figure 5:**
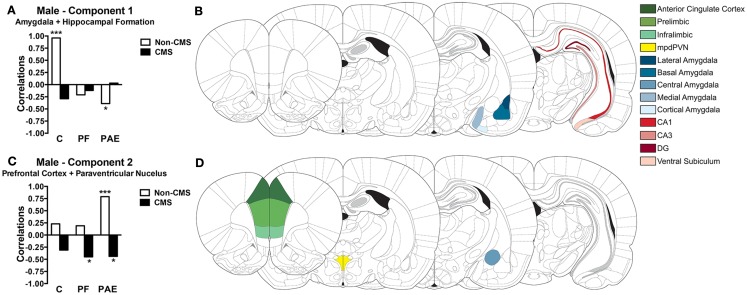
**Bars represent the correlation between the experimental group and the component scores for components 1 (A) and 2 (C) extracted from the CPCA for males**. Schematic figure highlighting the areas that contributes to each neural network **(B,D)**. Images were adapted from Ref. (46), ***p < 0.0001; *p < 0.05. C, control; PF, pair-fed; and PAE, prenatal alcohol exposed.

The experimental conditions (i.e., prenatal treatment and CMS exposure) accounted for 34.17% of the total variance in *c-fos* mRNA expression in males. PCA on this constrained variance (i.e., the predictable variance) revealed a two-component solution; Table 1 displays the component loadings for each of the 13 brain regions on each component. The first component explained 19.40% of the total variance and 56.78% of the predictable variance and was defined as *Amygdala* + *Hippocampal Formation* because the medial, cortical, and basal amygdala nuclei, and CA3 and CA1 hippocampal regions showed the highest loadings, with lesser contributions from the ventral subiculum, DG, and lateral amygdala (Figure 5B). The second component explained 11.26% of the total variance and 32.97% of the predictable variance and was defined as *Prefrontal Cortex* + *Paraventricular Nucleus* because the PrL, mpdPVN, and ACC showed the highest loadings, with lesser contributions from the IL and central amygdala (Figure 5D).

**Table 1:** **Component loadings for the predicted solution in males**.

Variables	*Amygdala* + *Hippocampal Formation*	*Prefrontal Cortex* + *Paraventricular Nucleus*
Medial amygdala	**0.65**	0.06
Cortical amygdala	**0.63**	0.09
CA3	**0.61**	0.17
Basal amygdala	**0.58**	0.04
CA1	**0.53**	0.09
Ventral subiculum	**0.48**	0.20
DG	**0.44**	0.24
Lateral amygdala	**0.37**	−0.17
Prelimbic	0.14	**0.59**
Paraventricular nucleus	0.12	**0.54**
Anterior cingulate	0.07	**0.54**
Infralimbic	0.22	**0.48**
Central amygdala	0.21	**−0.36**

*Values ≥0.30 are set in bold*.

Correlations between the subjects’ component scores and their experimental condition membership are displayed in Figure 5A for the *Amygdala* + *Hippocampal Formation network* and in Figure 5C for the *Prefrontal Cortex* + *Paraventricular Nucleus network*. The *Amygdala* + *Hippocampal Formation network* showed a significant positive correlation with the control non-CMS condition (*r* = 0.96, *p* < 0.0001), but a significant negative correlation with the PAE non-CMS condition (*r* = −0.39, *p* < 0.05). Furthermore, although not reaching statistical significance, the *Amygdala* + *Hippocampal Formation network* was also negatively correlated with the control CMS condition. As seen in Figure 5C, the *Prefrontal Cortex* + *Paraventricular Nucleus network* showed positive correlations with all non-CMS conditions; however only PAE non-CMS (*r* = 0.79, *p* < 0.0001) reached significance. Conversely, the *Prefrontal Cortex* + *Paraventricular Nucleus network* showed negative correlations with all CMS groups, with PAE (*r* = −0.44, *p* < 0.05) and PF (*r* = −0.45, *p* < 0.05) CMS conditions showing significant correlations.

#### Females

**Figure 6:**
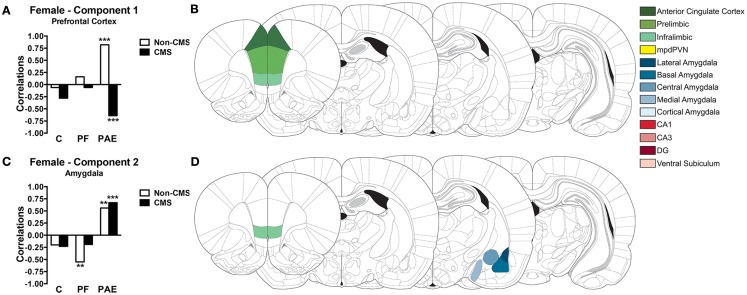
**Bars represent the correlation between the experimental group and the component scores for components 1 (A) and 2 (C) extracted from the CPCA for females**. Schematic figure highlighting the areas that contributes to each neural network **(B,D)**. Images were adapted from Ref. (46), ***p < 0.0001; **p < 0.01. C, control; PF, pair-fed; and PAE, prenatal alcohol exposed.

The experimental conditions accounted for 25.72% of the total variance in *c-fos* expression in females. PCA on this constrained (i.e., predictable) variance revealed a two-component solution (see Table 2 for component loadings). The first component explained 12.04% of the total variance and 46.81% of the predictable variance and was defined as *Prefrontal Cortex* because all mPFC subregions (ACC, IL, and PrL) contributed with the highest loadings to this component (Table 2; Figure 6B). The second component explained 9.14% of the total variance and 35.56% of the predictable variance and was defined as *Amygdala* because the majority of amygdala nuclei (central, lateral, medial, and basal) contributed with the highest loadings to this component; however, the IL also contributed to this component (Table 2; Figure 6D).

**Table 2:** **Component loadings for the predicted solution in females**.

Variables	*Prefrontal Cortex*	*Amygdala*
Anterior cingulate	**0.69**	0.20
Infralimbic	**0.62**	**0.35**
Prelimbic	**0.61**	0.24
Cortical amygdala	0.21	0.15
CA3	0.20	−0.09
DG	−0.23	0.01
Central amygdala	0.12	**0.60**
Lateral amygdala	−0.06	**0.56**
Medial amygdala	0.29	**0.34**
Basal amygdala	0.26	**0.31**
CA1	0.10	0.17
Paraventricular nucleus	0.00	−0.15
Ventral subiculum	0.03	0.03

*Values ≥0.30 are set in bold*.

Correlations between the subjects’ component scores and their experimental condition membership are displayed graphically in Figure 6A for the *Prefrontal Cortex network* and in Figure 6C for the *Amygdala network*. The *Prefrontal Cortex network* showed a significant positive correlation with the PAE non-CMS condition (*r* = 0.82, *p* < 0.0001). In contrast, the *Prefrontal Cortex network* showed a significant negative correlation with the PAE CMS condition (*r* = −0.64, *p* < 0.0001). Additionally, as seen in Figure 6C, the *Amygdala network* showed a significant positive correlation with the PAE group, independent of CMS condition (*r* = 0.56, *p* < 0.01 for PAE non-CMS and *r* = 0.67, *p* < 0.0001 for PAE CMS); however, this network was negatively correlated with the control group, again, independent of CMS condition. Furthermore, the *Amygdala network* showed a significant negative correlation with the PF non-CMS condition (*r* = −0.55, *p* < 0.01).

## Discussion

Stress and emotional regulation are dynamic and complex processes achieved by the fine coordination of interconnected brain regions, including, but not limited to, the amygdala, mPFC, hippocampus, and paraventricular nucleus of hypothalamus (PVN) (14–17, 51). The majority of these brain areas have dual functions, playing major roles in both the stress response and emotional regulation. Because of these dual roles and the intrinsic interconnectivity among these brain regions, dysfunction within or among structures in this neurocircuitry could result in dysregulation of the stress response and/or lead to mood and anxiety disorders. The present results indicate that, regardless of chronic stress later in life, PAE produces widespread alterations in neural activity within and between several brain areas of the stress/emotional neurocircuitry analyzed. Notably, male and female rats in the current study were differentially affected by PAE. Overall, PAE decreased neural activity within the amygdala and hippocampal formation in males and increased neural activity within the amygdala and mPFC in females. Exposure to CMS also resulted in a sexually dimorphic effect, as it reduced neural activity within the mPFC and PVN in PAE males, but reduced activity in all areas analyzed in controls. Conversely, CMS reduced neural activity only in the mPFC of PAE females and had no effects in control females. Importantly, these patterns of neural activity were reflected in the differential networks of brain activation for PAE and control male and female rats. Indeed, the CPCA revealed that, contrary to non-CMS PAE males, non-CMS control males showed a strongly active *Amygdala* + *Hippocampal Formation network*. Conversely, non-CMS PAE males showed a strongly active *Prefrontal Cortex* + *Paraventricular Nucleus network*. Among females, non-CMS PAE animals showed strongly active *Prefrontal Cortex* and *Amygdala networks*, which contrasts with the network activity observed in non-CMS controls. Of note, CMS also altered the networks of brain activation in PAE animals, as the pattern of activity within the *Prefrontal Cortex* + *Paraventricular Nucleus network* in PAE males and the *Prefrontal Cortex network* in PAE females switched from being active in the non-CMS animals to showing decreased activation in CMS animals. Overall, the changes in the network of brain areas following PAE could be implicated in the hyperresponsivity to stress and increased vulnerability to the development of psychopathologies observed among individuals prenatally exposed to alcohol (5, 9, 11–13, 22, 23).

### Amygdala

Clinical studies have consistently demonstrated that amygdala dysfunction is associated with mood and anxiety disorders (16, 17, 52, 53). These disorders are among the most prevalent mental health problems in children and adults exposed to alcohol during gestation (11–13). Animal models have supported the relationship between amygdala dysfunction and anxiety- and depressive-like behaviors (52–55). Specifically, the basal and lateral amygdala nuclei have been defined as part of the neural circuitry activated in animals exhibiting depressive-like behaviors (56, 57). Additionally, the central, lateral, and basal amygdala nuclei have a large population of neurons that express corticotropin-releasing hormone (CRH) and its receptors (58, 59); hyperfunction of these neurons can result in increased levels of anxiety-like behaviors (60, 61).

The present results indicate sexually dimorphic effects of PAE and/or CMS on neural activity of the amygdala nuclei. PAE females showed an increase in neural activity within the central and lateral amygdala nuclei in response to acute stress, regardless of whether they were exposed to CMS. Increased neural activity in the amygdala is consistent with the increased stress responsiveness as well as anxiety-/depressive-like behavior often seen in PAE females (5, 31, 32). However, vulnerability to anxiety-/depressive-like behavior is often greater in PAE animals following CMS than in their non-CMS counterparts, and under some conditions, non-CMS PAE animals may not differ from non-CMS controls. We found, for example, that while non-CMS PAE females were similar to non-CMS controls in time spent on the open arms of an elevated plus maze, exposure to CMS significantly reduced frequency of total open arm entries for PAE but not control females (31). These discrepancies in neural activity and behavior suggest that other brain areas besides the amygdala may be modulating anxiety-like behavior among PAE females. In males, PAE and pair-feeding prevented the increase in neural activity within the medial, cortical, and basal amygdala nuclei shown by control non-CMS animals. However, compared to controls, neither PAE nor CMS changed the neural activity of central and lateral amygdala nuclei in response to acute stress. These data are interesting in light of the finding that PAE males exposed to CMS show more robust increases in anxiety-like behavior in the elevated plus maze than PAE females (31), suggesting that the neural mechanisms involved in mediating the anxiety response among males may be dissociable from those in females. This is supported by our finding of networks of brain regions that were differentially associated with PAE and/or CMS in males and females in the current study, as discussed further below.

In addition to its well-characterized role in emotional processing, the amygdala also regulates different aspects of the neuroendocrine stress response (15, 62). In general, the central nucleus of the amygdala regulates the HPA response to systemic stressors, and integrates autonomic responses to psychogenic stressors; the basal, lateral, and the medial amygdala nuclei regulate the HPA response to psychological stressors (62). The increased neural activity within the lateral amygdala of PAE females reported here may, at least partially, underlie the hyperresponsivity to stress observed in these animals (5, 9, 22, 23).

### Hippocampal formation

The hippocampal formation is well known for its role in learning and memory, stress, and emotional regulation (63). However, the dorsal and ventral components of the hippocampal formation have different functions: the dorsal hippocampus is primarily involved in cognitive functions while the ventral hippocampus is more essential for stress and emotional regulation (63). For example, lesions to the ventral hippocampus result in decreased anxiety-like behaviors (63, 64), whereas increased activity in the ventral hippocampus is positively correlated with anxiety-like behavior (65, 66). Additionally, the hippocampal formation is consistently associated with an overall inhibition of the stress response (14, 15). Indeed, hippocampal lesions result in increased stress responses and, in some cases, increased basal levels of corticosterone (67). Stimulation of the CA3, DG, and ventral subiculum subfields, on the other hand, results in reduced glucocorticoid secretion (68). Interestingly, PAE is known to induce morphological changes that can lead to functional deficits in the hippocampus: studies in the clinical literature reveal reduced hippocampal volume in individuals exposed to alcohol during gestation (20, 21). In rats, PAE results in hypertrophy of mossy fibers (69), reduced numbers of dendritic spines (70), as well as impaired long-term potentiation (71, 72) and adult neurogenesis – primarily in males (73, 74). In agreement with these findings, our results indicate that PAE males show reduced neural activity in the hippocampal formation in response to acute stress when compared to controls. This deficit in neural activity suggests that the inhibitory action of the hippocampal formation may be reduced, which could contribute to the hyperresponsivity to stress, and ultimately to the increase in anxiety-like behaviors observed in PAE animals (31).

Interestingly, PAE results in no apparent changes in basal expression of glucocorticoid and mineralocorticoid receptors in the hippocampal formation of male animals (75–77), whereas in females, some studies indicate a reduction (77), whereas others report no change (75, 76) in basal expression of glucocorticoid and mineralocorticoid receptor mRNA. However, removal of the inhibitory feedback effects of corticosterone on the HPA axis by adrenalectomy unmasked alterations in basal HPA regulation not apparent in intact animals. Glucocorticoid receptor expression was differentially increased in the hippocampus of PAE males (75) whereas mineralocorticoid receptor expression was differentially increased in the hippocampus of PAE females compared to their control counterparts (75). Moreover, corticosterone replacement was shown to be ineffective at normalizing the adrenalectomy-induced increase in hippocampal mineralocorticoid mRNA levels in PAE males. Together, these results indicate that altered basal regulation of the HPA axis may play a critical role in the hyperresponsivity to stress observed in individuals exposed to alcohol during the prenatal period.

Our results also indicate that CMS reduced the neural activity in the hippocampal formation of control males. This is not surprising given the previous finding that CMS can reduce the number of hippocampal granule cells (78), which could be related to reduced survival of newborn cells (79).

### Medial prefrontal cortex

The mPFC also plays a dual role in modulating stress and emotional responses. Abnormal volume and activation of the PFC are commonly observed in individuals with anxiety and mood disorders (16, 52). Importantly, functional neuroimaging studies indicate that individuals exposed to alcohol during gestation show greater blood oxygen level-dependent (BOLD) responses in the PFC when performing a behavioral inhibition task (18), suggesting that the PFC could be responding differentially when challenged. In rodents, reduced function of the mPFC, induced by electrolytic lesions or temporary deactivation, results in variable effects: some authors report decreased anxiety-like behaviors (80–82) while others report increased anxiety-like behaviors (83, 84). These discrepancies on the effects of the mPFC on anxiety-like behavior may be due to the extent of the lesions/deactivation and on the heterogeneous functions of the mPFC subregions. However, overall dysregulation of the mPFC can lead to an increased predisposition to psychopathologies. Our results show that PAE and control females exhibit different mPFC responses to the acute stress. Despite the fact that mPFC neural activity is increased in PAE non-CMS females, their behavioral performance was not different from controls in the elevated plus maze (31). Conversely, the neural activity within the mPFC of PAE males was not different from controls; however, PAE increased the expression of anxiety-like behaviors in male rats (31). This dissociation between behavioral and mPFC neural outcomes likely reflects the complex nature of anxiety- and depressive-like disorders, and suggests that other brain areas besides the mPFC may play a role in supporting increased anxiety-like behaviors observed in PAE animals.

The mPFC also plays a major role in modulating the HPA response to stressful events. However, its role is complex due to the fact that the PrL and ACC subregions are involved in inhibition while the IL is involved in excitation of the HPA axis (15, 85–87). Regardless of these opposing roles, our results show that the neural activity within the PrL and ACC in PAE and control animals follows a pattern similar to that within the IL. Specifically, PAE non-CMS females showed a higher response to acute stress in all mPFC areas than their female counterparts, with CMS reducing this neural activity. For the PrL, it is possible that the reduced neural activity in PAE females exposed to CMS may be associated with a reduction in CRH mRNA expression within this area (77), suggesting that the inhibitory action of the PrL may be reduced following CMS, contributing to the hyperresponsivity to stress in PAE females. Similar to the PrL, the ACC is also associated with inhibition of the HPA axis (15, 85–87) and the reduced neural activity of the ACC in PAE females exposed to CMS could also contribute to the hyperresponsivity to stress in those animals. Despite an opposite role, the IL – which is involved in excitation of the HPA axis (15, 85–87) – also showed reduced neural activity in PAE females exposed to CMS, which suggests a possible alteration in the excitatory/inhibitory balance within the mPFC of PAE females.

### Medial parvocellular dorsal division of the paraventricular nucleus of hypothalamus

The mpdPVN is composed of a discrete set of neurons that integrate all direct and indirect stress-related inputs from the amygdala, mPFC, and hippocampal formation in order to launch an appropriate response to stress (15, 62). These neurons synthesize and secrete CRH, the primary regulator of the pituitary–adrenal axis (88). Our results indicate that, independent of sex, PAE, and control rats showed similar neural activity in the mpdPVN in response to the acute stress of exposure to the elevated plus maze. However, when facing a noxious stressor, such as shock, or a major challenge to HPA regulation, such as adrenalectomy, PAE has been shown to induce enhanced neural activity within the PVN (75, 89). Studies have demonstrated that exposure to the elevated plus maze induces small increases in neural activity within the PVN of control animals when compared to the neural activity of animals exposed to shock (34). These results suggest that when PAE animals are tested under basal conditions, or face a mild stressor such as the elevated plus maze, the PVN is capable of responding appropriately; however, when faced with a more severe stressor such as shock, the PVN launches an inappropriately large response.

### Constrained principal component analysis

The univariate results above indicate that PAE is associated with changes in neural activity across a wide range of brain regions; however, it is important to examine not only individual brain regions, but also how these regions interact to form networks and whether these networks might be differentially altered by PAE and/or CMS. In order to examine this possibility, we employed CPCA, which allowed for the identification of combinations of brain regions that were affected by PAE. CPCA is an ideal statistical technique for examining networks of brain regions that are related to one or more variables of interest (in the current study, prenatal treatment and CMS exposure) because networks are defined only from the portion of the overall variance that is predictable from these independent variables. As such, it was possible to identify networks of brain regions that were associated with PAE (and CMS) in the current study. Comparison between the univariate and multivariate results shows several similarities in terms of the brain regions affected by PAE and CMS; however, the aggregation of these brain regions into networks through CPCA facilitates interpretation.

#### Networks regulating stress and emotion in males

In males, CPCA revealed two functional networks that were collectively responsible for 34.17% of the total variation. The first network included the majority of the amygdala nuclei (medial, cortical, basal, and lateral) and all of the hippocampal formation areas analyzed (CA1, CA3, DG, and ventral subiculum). This *Amygdala* + *Hippocampal Formation network* was strongly active in control animals that were not exposed to CMS, but showed decreased activation in controls subjected to CMS. These findings suggest that the *Amygdala–Hippocampal Formation network* is critically involved in stress and emotional regulation in control males, and that exposure to chronic unpredictable stress induces functional changes in this network by preventing typical neural activation of the amygdala and hippocampal formation in response to acute stress. Dysregulation in this network may partially explain the increased susceptibility to the development of psychopathologies and abnormal stress responses in animals subjected to CMS (90–93). Importantly, in contrast to controls, the *Amygdala* + *Hippocampal Formation network* showed decreased activation for PAE non-CMS animals, indicating that PAE may alter the typical neural activity in these areas such that PAE animals in the non-CMS conditions look similar to controls in the CMS condition.

The second network extracted from the CPCA included all of the mPFC areas analyzed (PrL, ACC, and IL) and the mpdPVN. This *Prefrontal Cortex* + *Paraventricular Nucleus network* was activated to some degree in all prenatal groups in the non-CMS condition, although activation of this network was statistically significant only in the PAE non-CMS group. These data suggest that, independent of prenatal treatment, the mPFC and the mpdPVN form a network of structures that work together in the regulation of stress and emotion in males, but that PAE animals engage this network to a greater degree than control animals. This phenomenon could be a compensatory mechanism for the reduced involvement of the *Amygdala* + *Hippocampal Formation network*, as indicated by the negative correlation for PAE non-CMS males with this network. Additionally, all prenatal groups subjected to CMS in adulthood showed reduced activity in the *Prefrontal Cortex* + *Paraventricular Nucleus network* following acute stress. This switch between positive and negative associations with this network following exposure to CMS highlights the vulnerability of the mPFC and mpdPVN to chronic and unpredictable stress in adulthood. Indeed, the preclinical literature has demonstrated that the PFC is extremely susceptible to the effects of CMS, as chronic stress results in morphological alterations to mPFC neurons, including retraction of the apical dendritic branches and spine loss in layer II/III neurons (94–96). Additionally, stress-related morphological changes in the mPFC have been associated with increased anxiety- and depressive-like behaviors (90, 97) as well as with dysfunction in attentional set shifting and working memory (98, 99).

#### Networks regulating stress and emotion in females

Similar to males, CPCA indicated two functional neural networks in females. However, these networks were more restricted and only included major subdivisions of the same brain regions. The first network (*Prefrontal Cortex*) included all areas of the mPFC (ACC, IL, and PrL); the second network (*Amygdala*) included four nuclei of the amygdala (central, lateral, medial, and basal) and the IL. Together, these two functional neural networks in females accounted for 25.72% of the total variation, in contrast to 34.17% accounted for by the two functional networks in males.

The *Prefrontal Cortex network* was strongly activated in PAE non-CMS females, which contrasts with the decreased activity observed among control non-CMS females. Moreover, exposure to CMS resulted in a negative association for PAE females with the *Prefrontal Cortex network*, whereas there was no significant change in activation of this network in control females. These results indicate that PAE females rely on the *Prefrontal Cortex network* more than control females for stress and emotional regulation. This abnormal engagement of the *Prefrontal Cortex network* in response to acute stress is also observed in PAE males and indicates that alcohol exposure during the gestational period results in dysregulation of mPFC function in both sexes. Additionally, our results suggest that the mPFC of PAE females is more susceptible to the effects of CMS when compared to controls. The switch between positive and negative associations for the *Prefrontal Cortex network* following exposure to CMS highlights the vulnerability of the mPFC to chronic and unpredictable stress.

Similarly, the *Amygdala network*, which also contains the IL region, showed decreased activity in controls, independent of CMS condition, but was significantly positively activated in PAE females independent of CMS. As the amygdala and the IL subregion of the mPFC activate the HPA axis (14, 15), this abnormal increased activity of the *Amygdala network* following PAE may underlie, at least in part, the hyperresponsivity to stress observed in those animals. Additionally, the activity of the *Amygdala network* in response to acute stress in control and PAE females was not altered by CMS, indicating that, regardless of the level of activity, the amygdala of controls and PAE females is more resistant to chronic and unpredictable stress.

### Pair-feeding

The PF group is used to control the reduced food intake typically observed in alcohol-consuming dams. However, pair-feeding is an imperfect control procedure as it cannot control for the effects of alcohol on absorption and utilization of nutrients. Additionally, despite receiving optimal nutrition during pregnancy (43), the PF dams are underfed compared to the *ad libitum*-controls, as they receive a reduced ration matched to that of an alcohol-consuming partner. As a result, they are hungry, and typically consume their entire daily food ration within a few hours of presentation, remaining food deprived for the rest of the day. This pair-feeding regimen is thus a mild prenatal stressor, and its effects on offspring behavioral and physiological responsiveness represent, at least partially, an effect of stress above and beyond the nutritional aspect of receiving a reduced food ration. Our results suggest that PF males showed a pattern of neural activity within the amygdala and hippocampal formation similar to PAE males, while the pattern of neural activity within the mPFC and mdpPVN was similar to both PAE and controls. For females, on the other hand, the pattern of neural activity for PF animals was similar to the pattern displayed by the control group. Taken together, these results suggest that the males may be more susceptible to the effects of pair-feeding than females. However, the only effect exclusively observed in PF animals was a significant negative correlation of the *Amygdala network* with the non-CMS PF female condition, which suggests that stress and emotional regulation rely less on the amygdala in PF than PAE or control females.

### Summary and conclusion

The results presented here build on and expand our knowledge of the differential neural regulation of stress and emotion in individuals exposed to alcohol during gestation. Importantly, despite a relatively low number of animals in one of the experimental groups, the use of CPCA provided a powerful approach for assessing global changes in functional neural networks in PAE compared to control animals, allowing us to go beyond simple assessments of changes in individual brain areas. Indeed, our results highlight how PAE and control males and females recruit different brain networks in the regulation of stress responses and emotion. Specifically, CPCA indicates that while control males rely more on the amygdala and hippocampal formation, PAE males rely more on the mPFC and mpdPVN when facing a stressful situation. In contrast, the functional neural networks underlying stress and emotional regulation in females are more restricted, as PAE females rely primarily on the mPFC and amygdala. Additionally, our results indicate that CMS differentially affected the neural networks regulating stress and emotion in PAE and control animals. Indeed, exposure to CMS reduced the activity of the *Amygdala* + *Hippocampal Formation network* in control males, but reduced the activity of the *Prefrontal Cortex* + *Paraventricular Nucleus network* in PAE males. For females, CMS only reduced the activity of the *Prefrontal Cortex network* in PAE animals. Together, our results suggest that PAE, regardless of its association with CMS, results in a sexually dimorphic dysregulation of the neurocircuitry that underlies stress and emotional regulation, which may be implicated in the stress hyperresponsivity and increased vulnerability to anxiety and depressive disorders observed among individuals exposed to alcohol during gestation (5, 9, 22, 23, 31). Finally, understanding the underlying neural mechanisms of deficits induced by PAE is a crucial step toward the establishment of specific strategies for treating resultant psychopathologies in these individuals.

## Conflict of Interest Statement

The authors declare that the research was conducted in the absence of any commercial or financial relationships that could be construed as a potential conflict of interest.
